# A Boron Delivery Antibody (BDA) with Boronated Specific Residues: New Perspectives in Boron Neutron Capture Therapy from an In Silico Investigation

**DOI:** 10.3390/cells10113225

**Published:** 2021-11-18

**Authors:** Alessandro Rondina, Paola Fossa, Alessandro Orro, Luciano Milanesi, Antonella De Palma, Davide Perico, Pier Luigi Mauri, Pasqualina D’Ursi

**Affiliations:** 1 Institute for Biomedical Technologies, National Research Council (ITB-CNR), 20054 Segrate (MI), Italy; alessandro.rondina@itb.cnr.it (A.R.); alessandro.orro@itb.cnr.it (A.O.); luciano.milanesi@itb.cnr.it (L.M.); antonella.depalma@itb.cnr.it (A.D.P.); davide.perico@itb.cnr.it (D.P.); 2 Department of Pharmacy, Section of Medicinal Chemistry, School of Medical and Pharmaceutical Sciences, University of Genoa, 16132 Genoa, Italy; paola.fossa@unige.it

**Keywords:** Boron Neutron Capture Therapy, 4-borono-L-phenylalanine, Boron Delivery Antibody strategy, docking, molecular dynamics

## Abstract

Boron Neutron Capture Therapy (BNCT) is a tumor cell-selective radiotherapy based on a nuclear reaction that occurs when the isotope boron-10 (^10^B) is radiated by low-energy thermal neutrons or epithermal neutrons, triggering a nuclear fission response and enabling a selective administration of irradiation to cells. Hence, we need to create novel delivery agents containing ^10^B with high tumor selectivity, but also exhibiting low intrinsic toxicity, fast clearance from normal tissue and blood, and no pharmaceutical effects. In the past, boronated monoclonal antibodies have been proposed using large boron-containing molecules or dendrimers, but with no investigations in relation to maintaining antibody specificity and structural and functional features. This work aims at improving the potential of monoclonal antibodies applied to BNCT therapy, identifying in silico the best native residues suitable to be substituted with a boronated one, carefully evaluating the effect of boronation on the 3D structure of the monoclonal antibody and on its binding affinity. A boronated monoclonal antibody was thus generated for specific ^10^B delivery. In this context, we have developed a case study of Boron Delivery Antibody Identification Pipeline, which has been tested on cetuximab. Cetuximab is an epidermal growth factor receptor (EGFR) inhibitor used in the treatment of metastatic colorectal cancer, metastatic non-small cell lung cancer, and head and neck cancer.

## 1. Introduction

One of the greatest and still unsolved challenges in cancer therapy is specifically targeting tumor cells without damaging the surrounding healthy cells. Chemotherapy produces severe side effects to normal cells due to toxicity, and radiation therapy causes destruction of the neighboring safe tissues and of those crossed by the radiation beam. Boron Neutron Capture Therapy (BNCT) is an emerging tumor cell-selective radiotherapy based on a nuclear reaction that occurs when the isotope boron-10 (^10^B) is radiated by low-energy thermal neutrons or epithermal neutrons, triggering a nuclear fission response that produces an alpha particle (4He) and a lithium-7 (7Li) nucleus with a high Linear Energy Transfer (LET) [1]. The LET particles have a path length of 5−10 μm; this is very close to the diameter of a cell, thus limiting their destructive effects on the boron-containing cells. To obtain an appropriate generation of radiation from the boron neutron capture reaction, which means a successful therapy, high amounts of boron (at least 10^9^ boron atoms per cell) have to be accumulated in cancer cells. Only two compounds are currently used in clinical applications for BNCT, namely ^10^B-boronophenylalanine (BPA) and sodium mercaptoundecahydro-closo-dodecaborate (BSH) [2].

In recent years, accelerator-based neutron sources have been proposed; they are more compact and less expensive than a reactor, and can be installed in hospitals permitting an increase in clinical trials [3]. In 2020, the Ministry of Health, Labor, and Welfare of Japan approved the world’s first medical boron drug and devices for BNCT, specifically for the treatment of locally unresectable recurrent or unresectable advanced head and neck cancers (HNC), based on borofalan (Steboronine^®^) and the accelerator neutron source [4,5].

In addition to the optimization of epithermal neutron spectrum of accelerator-based BNCT, which has become comparable to nuclear reactor-based BNCT [6], of primary importance is the improvement of the boron carriers (such as nanoparticles and proteins) in increasing the uptake into target cells [7,8,9]. Moreover, the heterogeneity of tumors and new boron carriers should be accounted for, so as to deduce *a priori* their distribution in the body and their concentration in specific tissues [10].

In order to gain a more selective and efficient therapy, we were interested in targeting only specific proteins located in/on cancer cells. In the past, boronated Epidermal Growth Factor EGF was chemically linked to a heavily boronated polyamidoamine dendrimer (BD) [11,12]. However, despite the mild reaction conditions used to conjugate EGF to the BD, a significant decrease in the K_A_ of the bioconjugate was observed, probably due both to EGF conformational changes and to steric hindrance by the bulky BD groups, which impaired EGF binding to the epidermal growth factor receptor, EGFR [13].

In this context, we developed a computational protocol to evaluate if a specific monoclonal antibody with boronated residues was still capable of recognizing its specific target protein in/on tumor cells. This computational approach is based on reduced antibody conformational changes and steric hindrance interactions with the biological target, to maintain a significant binding affinity between the two proteins. The protocol is generalizable and may be applied to any monoclonal antibody used in cancer therapy. In the present work, cetuximab—a chimeric monoclonal antibody capable of inhibiting EGFR and decelerating tumor growth—is discussed as a case study. Cetuximab is used for the treatment of metastatic colorectal cancer, metastatic non-small cell lung cancer, and head and neck cancer. Of note, the amount of EGF receptor increases up to 10^6^ times on tumor cells than on normal cells, demonstrating a significant accumulation of cetuximab [14,15,16]. EGFR is a transmembrane glycoprotein that belongs to the ErbB receptor family [17]. Since EGFR activation induces macropinocytosis, it is suitable for BNCT, which requires high selectivity to maximize ^10^B concentrations in cancer cells. The efficient cellular uptake of boron atoms inserted into the antibody is in fact guaranteed.

The results obtained within this new approach will be discussed in light of their potential applications in therapy.

## 2. Materials and Methods

### 2.1. Pipeline Description

The pipeline has been developed to identify (a) the best candidates from a subset of boron-containing ligands obtained from the literature and DrugBank (see Section 2.2) and (b) the most suitable residues to be boronated. Based on the ligand scaffold similarity with side chains of amino acids, we selected 4-borono-L-phenylalanine and the L-enantiomer of cis-1-amino-3-borono-cyclopentanecarboxylic acid for their similarity with tryptophan, histidine, phenylalanine, and tyrosine residues. In the first step to evaluate the most suitable residues to be modified/boronated on the protein, all histidine and tyrosine residues were mutated into glycine and then into alanine. In this way, we created two subsets of cavities to be explored for boronation. Two selected boron ligands were then simplified into fragments and used as exploring probes in docking studies using AutoDock Vina [18] to identify a pool of the best cavities capable of hosting boronated side-chain residues. The docking results were automatically filtered by a Python script based on energetic ranking and steric overlapping between original residues and modified ligand, in terms of distance and directionality.

Validation of the results via visual inspection was also performed, which took into account not only affinity score levels but also ligand orientation, degree of overlap, and ligand distance from the respective side chains of the mutated residues.

Biophysical feature characterization based on functional group, spatial constraints, and the chemical properties of side chain groups allowed us to identify the candidate residues for boronation.

Molecular dynamics (MD) simulations with the native and the modified mAb were then performed and compared to evaluate whether the new mutation was acceptable and ensure it did not impact protein folding.

### 2.2. Fragment Probe for Docking

Molecules from the BNCT literature and a subset of 75 drugs containing boron atoms from DrugBank [19] were taken into account. The two best candidates, 4-borono-L-phenylalanine and L-enantiomer of cis-1-amino-3-borono-cyclopentanecarboxylic acid, were selected. Three fragment probes, namely phenylboronic acid, *p*-toluene boronic acid, and cyclopentylboronic acid, were generated. The 3D structure of the fragment probes was then built in mol2 format from the SMILE linear representation, using Ligprep. Molecular charges were then computed with Epik under unspecified pH conditions. Finally, the pdbqt file for the docking procedure was created with Autodock MGLTools. The boron atom was converted into a carbon atom, which best approximates boron and is the most commonly used substitute in computational studies, because boron is not parametrized in Autodock Vina. Adjustment to the lengths and bond angles between connected boron and atoms were carried out according to measurements provided by scientific literature [20]. In this way, the final ligands retained all the geometric structural characteristics and electrical charges of a molecule containing a boron atom (Supplementary Figure S1).

### 2.3. Case Study of Cetuximab Fab

We downloaded the crystallographic structure of cetuximab Fab (PDB ID: 1YY8) from the Protein Data Bank (PDB). The cetuximab residues best mimicked by the probes, for both their structural and chemico-physical features, were Phe, Tyr, Trp, and His. Each residue was mutated to Gly and subsequently to Ala. We selected chain A and chain B as light and heavy chains, respectively. Any mutation around the binding site with the EGFR membrane protein was excluded from mutation, as this interaction should be maintained for the desired antibody selectivity and activity. Docking simulations were carried out for each proposed mutation. Results were analyzed via visual inspection and a Python script, selecting the best residues to be mutated, taking into account not only affinity score levels but also orientation, degree of overlap, and ligand distance from the respective side chains of the mutated residues. Four candidate residues for substitution were identified: three located on the light chain (chain A Tyr140, chain A Tyr173, and chain A Tyr186) and one located on the heavy chain (chain B Tyr200). The impact of mutation on antibody structure stability and folding was evaluated through MD simulations on both mutated and native proteins for comparison. Procedural steps are described in the following subsections.

### 2.4. Fab Mutagenesis

Phe, Tyr, Trp, and His residues of cetuximab were mutated to Gly and subsequently to Ala using a wizard tool by Pymol 2.3.4 (PyMOL Molecular Graphics System, DeLano Scientific LLCSouth, San Francisco, CA, USA), consequently obtaining eight different mutated structures.

### 2.5. Docking Analysis

The eight structures were prepared for docking using Autodock MGLTools: water molecules and ions were removed, hydrogen atoms were added, and charges were assigned. Blind docking was performed between each of the three fragment probes and the mutated cetuximab proteins using AutoDock Vina. Phenylboronic acid, *p*-toluene boronic acid, and cyclopentylboronic acid were docked on modified cetuximab where Gly or Ala replaced Phe, Tyr, Trp, and His. Grid box parameters were set to a size of 56 × 64 × 88 Å^3^ and to a coordinate center of (32.771, 37.27, 32.376 Å). Exhaustiveness was set to the default value and the energy range was set to 4. The results obtained were filtered based on energetic ranking and steric overlapping between the original residues and the probes, in terms of distance, directionality, and steric clash by visual inspection with Pymol 2.3.4 (PyMOL Molecular Graphics System, DeLano Scientific LLCSouth, San Francisco, CA, USA) and a specific Python 3.8 script, implemented to derive the distance between a reference carbon atom on the original residue and the corresponding carbon atom on the probe (details are reported in Results, Section 3.2).

### 2.6. Boronated Amino Acid Residue Parametrization

To obtain the topological file for the boronated residue to be used in MD simulations, a mutated residue library and force field parameters were used. The cetuximab 3D structure was downloaded and processed through pdb4amber (AMBER Software, California, San Francisco, CA, USA), a script included in Amber. In the absence of starting coordinates to create a new boronated residue (BPA), the native coordinates of the backbone Tyr residues were extracted from the antibody to constitute the reference coordinates. The N- and C-terminal residues were reconstituted and the side chain oxygen was replaced by the functional group B(OH)_2_. Bond lengths and angles for the B(OH)_2_ moiety were obtained from experimental measurements [20]. Charges for the modified residue were calculated using the Epik tool, Schrodinger [21].

The ac file template for the prepgen [22] program was manually edited for the modified residues. The template was used to remove the excess atoms at the N- and C-terminals while the residue was ready to connect with the other amino acids in the antibody. Then, it was used to generate the prepin file. The latter was processed by tleap to generate the modified residue fcrmod file [23,24]. Once the new parameterized residue was obtained, the original tyrosines were replaced with the new mutated residue within the pdb files, called BPA. The new files were then uploaded to tleap to create topological and coordinate files useful in MD simulations.

### 2.7. MD Simulations

To study the dynamic behavior of modified residues within the protein context, five different MD simulation analyses were performed: one simulation for the native protein and then four simulations for the boronated proteins, one for each singular mutation. The MD simulations were carried out with the Amber18 Molecular Dynamics package [22,25] using the ff14SB force fields and parameters file of the modified residues described in the previous paragraph. The simulations were carried out with TIP3P water models in octahedral boxes; they were neutralized with counter ions and a salt concentration of 150mM was maintained based on the number of water molecules present in the model and on the charge of the solute [26]. Subsequently, an energy minimization of the system was carried out in four consecutive steps of 5000 steps with steepest descendent method to allow the system to stabilize gradually. Starting from a completely restrained system, except for the hydrogen atoms (restraint wt = 2.0), restraint was gradually removed, first from the water molecules (restraint wt = 1.0) and then from the native protein residues (restraint wt = 0.5). Finally, all restraints were released, with a cutoff for non-bonded interactions of 8 Å. The system was then heated in an NVT ensemble in Langevijn thermostat in two consecutive steps of 100,000 steps each, first from 0 K up to 200 K, and then up to 300 K. The system was then equilibrated in the NPT ensemble for 3 ns. Ultimately, each system was subjected to a simulation of 100 ns.

## 3. Results

### 3.1. Pipeline Description

**Figure 1:**
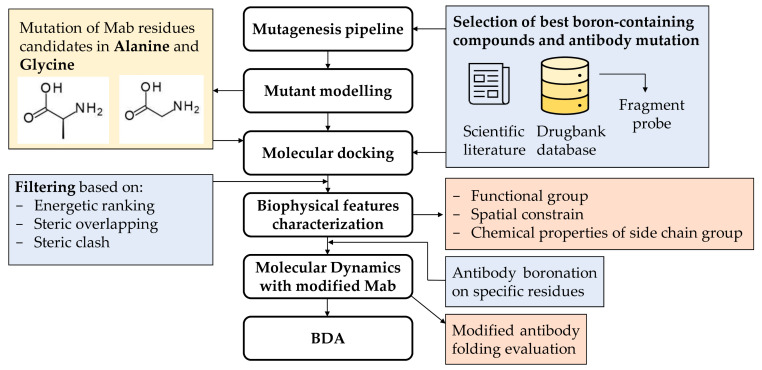
The BDA pipeline.

We developed a BDA (Boron Delivery Antibody) strategy, which improves the potential of monoclonal antibodies applied to BNCT therapy by identifying the antibody residues that can be replaced by a boronated analogue. The complete scheme of the computational procedure is shown in Figure 1. The pipeline has a modular design for the identification of the best amino acids that could be substituted by a boronated analogue, without impairment of the monoclonal antibody folding and its target protein recognition. The following main steps are discussed here.

#### 3.1.1. Selection of Best Boronated Compounds and Antibody Mutation

**Figure 2:**
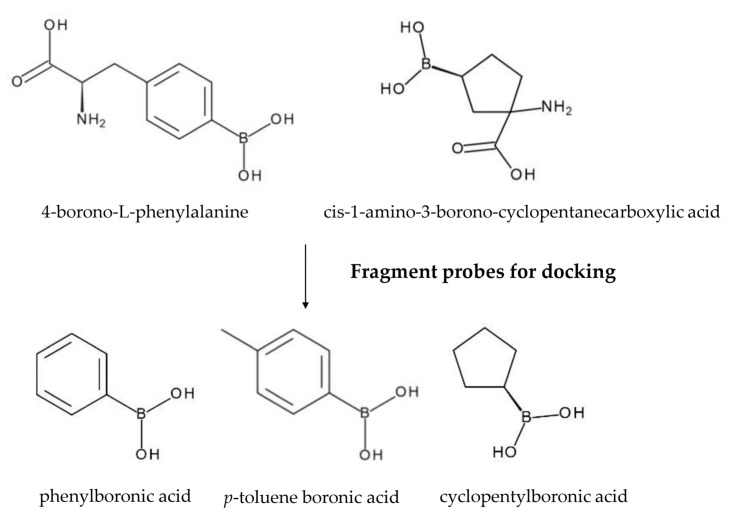
Chemical structures of the best boronated compounds identified and their fragments.

In a preliminary step, the fragment probes that best mimicked the chemico-physical features of some antibody amino acid residues were identified. A dataset of drugs was assembled from the BNCT literature and DrugBank boronated compounds, obtaining 75 molecules. Among these, 4-borono-L-phenylalanine and L-enantiomer of cis-1-amino-3-borono-cyclopentanecarboxylic acid, both already used in BNCT, were selected for their optimal scaffold similarity with Phe, Tyr, Trp, and His residues. From the two scaffolds, three fragments were generated. In the case of 4-borono-L-phenylalanine, the α- and β-carbon were removed and *p*-toluene boronic acid and phenylboronic acid were obtained, respectively. In the case of cis-1-amino-3-borono-cyclopentanecarboxylic acid case, the amino and the carboxyl groups were removed, thereby obtaining cyclopentylboronic acid (Figure 2).

To represent the 3D structure of the fragment probes, we used measurements provided by scientific literature. Lengths and bond angles between boron and atoms connected to it were properly set (Figure S1). In this way, the final ligands displayed all the geometric structural characteristics and electrical charges of a molecule containing a boron atom.

Each antibody residue able to mimic the chemico-physical properties of the probe fragments was mutated to Gly and then to Ala, in order to check whether the probe/candidate ligand side chain was capable of repositioning itself exactly in the region previously occupied by the side chain of the native residue. To evaluate the impact of the residue β-carbon atom in influencing the fragment probe pose, Gly mutation was additionally included to the Ala scanning mutation. The two mutations created two sets of cavities, further subdivided into four cavity subsets, one for each of the four mutated amino acid residues (Phe, Tyr, Trp, and His).

#### 3.1.2. Molecular Docking

**Figure 3:**
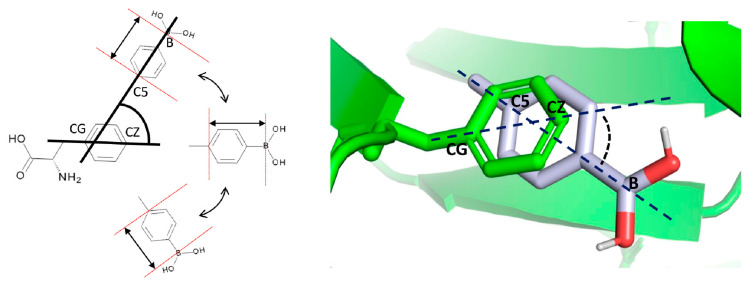
Evaluation of distance and orientation of each fragment with respect to the native residue by a Python script. Left: schematic representation of the different angles in which a docking pose can be located with respect to the reference residue; the residue vector (CG to CZ) and the ligand vector (C5 to B) serve as references for the calculation of the angle between them. Right: concrete example of the angle calculation between the Tyr residue and the *p*-toluene boronic acid ligand pose.

The three fragment probes were used to explore the eight cavity subsets via blind docking simulations. For each probe and for each subset, all poses obtained were analyzed by a Python script. The tool selected the best residues to be mutated based on energetic ranking, steric overlapping between the fragment probe and the native residue in terms of distance and directionality, and steric clashes. In Figure 3, chain A Phe 62 (from the Phe→Gly model derived from the cetuximab case study) is depicted as an example of pose evaluation based on distance and directionality. Probe orientations were evaluated by computing the angle between the reference vectors Phe@CB→CZ and Lig@C5→B.

The selected residues to be mutated were analyzed via visual inspection to further check their similarity with the probes in terms of structural and physical properties (H-bond ability, steric hindrance, and planarity).

#### 3.1.3. Antibody Boronation on Specific Residues

Each of the most promising amino acid residues identified by docking studies was modified into a boronated residue, based on the probes already selected. The generation of the new boronated residue took place starting from the initial coordinates of the α-carbon of the candidate residue. Since the boron atom is not parameterized in Amber18 force field, it was necessary to add the proper parameters and generate the corresponding residue topological file and coordinate file for the subsequent simulations (see the Materials and Methods section for details, Supplementary Figures S2 and S3 and Tables S1–S5).

#### 3.1.4. Modified Antibody Folding Evaluation

To evaluate the modified monoclonal antibody folding in comparison with the native folding, MD simulations were performed. In fact, it is necessary to preserve the original protein folding to retain the antibody functionality; thus, the new boronated residues should not cause folding alterations. RMSD and RMSF parameters were then calculated to check whether there were any alterations in the mutated protein stability compared to the wild-type. Subsequently, H-bond analysis allowed us to ascertain if the new residues maintained the native H-bond network. Finally, cluster analysis let us identify the most likely conformation of the modified monoclonal antibody by comparison with the native.

### 3.2. Case Study

**Figure 4:**
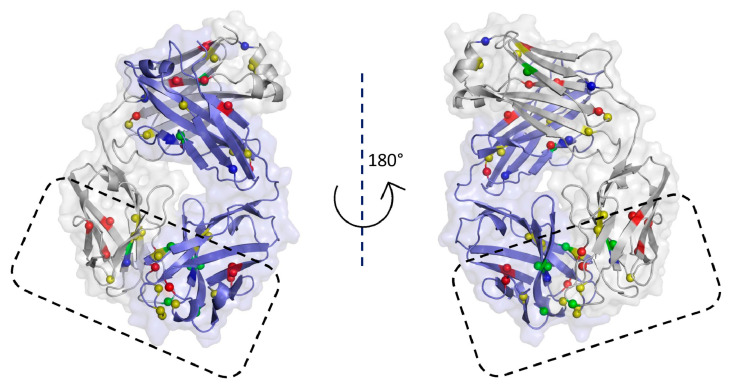
Cetuximab Fab: spheres indicate the location of Tyr (yellow), Trp (green), Phe (red), and His (blue) residues that were mutated into Gly and Ala. The contact area with the receptor, enclosed by a box, was not considered in this study.

Cetuximab, a monoclonal antibody capable of inhibiting epidermal growth factor receptor (EGFR), was selected as a case study to test our strategy and was mutated for delivering boron atoms. The XRay structure of cetuximab Fab (PDB id: 1YY8) alone and bound to the EGFR (PDB id: 1YY9) receptor were retrieved from PDB [27]. Both the heavy and the light chains of cetuximab participate in the interaction with the complementarity determining regions (CDRs) of the Fab fragment. The binding surface of the Fab fragment is rich in tyrosine and tryptophan, residues mimicked by the chemico-physical features of the probe fragments used in the docking. As a consequence, only the residues not involved in the interaction with the receptor were mutated by us to Gly and Ala (Figure 4), producing for each residue type (namely Phe, Tyr, Trp, and His) eight cavity subsets to be explored by different fragment probes as potential binding cavities.

In detail, when Phe, Tyr, Trp, and His were substituted with Gly or Ala, 16, 19, 8 and 6 cavities were obtained, respectively. In Figure 4, the mutated residues are accentuated using balls of different colors.

### 3.3. Docking Results

From the docking results, we only considered fragment probes poses localized inside the cavities left from the mutation of Phe, Tyr, Trp, and His into Gly and Ala.

**Figure 5:**
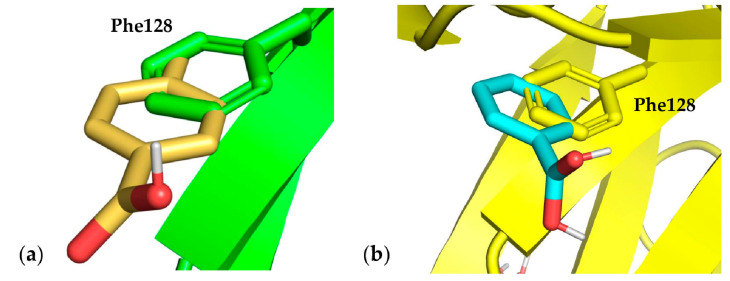
Best fragment poses for Phe mutation. (**a**) Docking pose number 2 for *p*-toluene boronic acid fragment probe in comparison with the native residue chain B Phe128, mutated to Gly; (**b**) Docking pose number 1 for phenylboronic acid fragment probe in comparison with the native residue chain B Phe128, mutated to Ala.

Phe residues were mutated into Gly and Ala in chain A Phe 21, 62, 71, 98, 116, 118, 139, and 209; and in chain B Phe27, 63, 79, 80, 106, 128, 152, and 172. The best performing residues were chain A Phe209, chain B Phe128, and chain B Phe152, with energy scores ranging from −5.60 to −4.20 (see Supplementary Tables S6–S9). Chain B Phe128 showed a close superimposition of its side chain when compared with the best pose of probe *p*-toluene boronic acid. In fact, in this case, the cavity generated by the Gly mutation was able to allow the fragment probe aromatic ring to occupy the native Phe side chain space and accept the boronic acid moiety (Figure 5).

**Figure 6:**
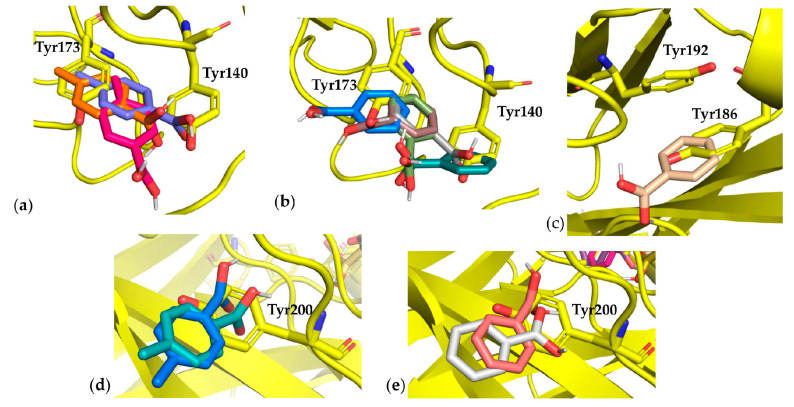
Best fragment poses for Tyr mutation. (**a**) Docking pose numbers 3, 4, and 7 for *p*-toluene boronic acid fragment probe in comparison with the native residues chain A Tyr173 and 140, mutated to Gly; (**b**) Docking pose numbers 4, 5, 7, 9, and 20 for phenylboronic acid fragment probe in comparison with the native residues chain A Tyr173 and 140, mutated to Ala; (**c**) Docking pose number 10 for phenylboronic acid fragment probe in comparison with the native residues chain A Tyr192 and 186, mutated to Ala; (**d**) Docking pose numbers 11 and 16 for phenylboronic acid fragment probe in comparison with the native residue chain B Tyr200, mutated to Ala; (**e**) Docking pose numbers 11 and 14 for *p*-toluene boronic acid fragment probe in comparison with the native residue chain B Tyr200.

Concerning Tyr, 19 residues were mutated to Gly or Ala in chain A Tyr36, 50, 86, 87, 140, 173, 186, and 192; and in chain B Tyr32, 59, 93, 94, 101, 102, 104, 108, 151, 182, and 200. Most of the best poses showed energy values spanning from −5.80 to −5.20 for Gly and from −5.30 to −4.60 for Ala (see Supplementary Tables S10–S13). Among these, mutations in chain A Tyr140, 173, 186 and 192 produced very interesting results, obtaining the best energy scores both in Gly and Ala mutations. Since the native Tyr residues are neighbors in both cases, the fragment probes occupied the cavity generated by mutations in different ways (Figure 6). Regarding Tyr 140–173, fragment poses partially overlapped on both the native residues, while in the case of Tyr186–192, the cavity created by the mutation was smaller and the fragment probe only overlapped with Tyr186 (Figure 6).

In both mutations, chain B Tyr200 fragment probe poses showed an opposite directionality (according to our settings) in overlapping with the native residue, resulting in discarding this mutation.

**Figure 7:**
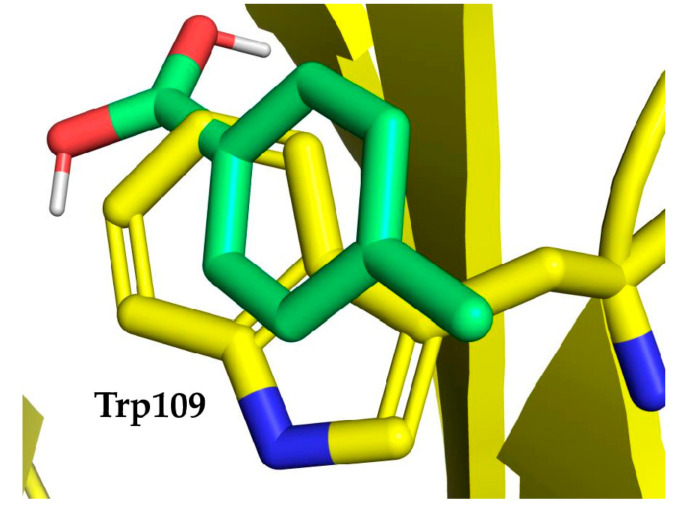
Fragment probe pose in comparison with the native residue—the case of Trp. Docking pose number 8 for *p*-toluene boronic acid fragment probe in comparison with the native residue chain B Tyr109, mutated to Gly.

Concerning Trp mutations, eight cavities were explored. Fragment probe poses for both Gly and Ala mutations showed similar energy scores, ranging from −6.70 to −5.70, but did not correctly match the Trp side chain orientation, according to our settings (Figure 7). Only in the case of pose number 8 obtained with *p*-toluene boronic acid and chain B Trp109 Ala mutation did the fragment probe result in being satisfactorily oriented with respect to the hydrophobic moiety of the native residue side chain. However, the polar moiety did not overlap with the polar nitrogen of the indole Trp ring (Figure 7). On this basis, we discarded the possibility of boronating Trp residues (Supplementary Tables S14–S17).

When His was mutated into Gly or Ala, fragment probe poses obtained with phenylboronic acid and *p*-toluene boronic acid were located outside the cavities derived from mutation (Supplementary Tables S18–S21).

The cyclopentil boronic acid probe, although having a partial structural similarity with His and Trp and, even lower, with Phe and Tyr, demonstrated the worst performance. In fact, it positioned itself outside the cavities generated by the mutations.

In conclusion, from the above reported docking results and their analysis, the best native residues were predicted to be boronated chain A Tyr140, chain A Tyr173, chain A Tyr 186, and chain B Tyr200.

### 3.4. Monoclonal Antibody Folding Evaluation Using MD Simulations

Finally, to evaluate whether the boronated residues are able to keep the wild type monoclonal antibody folding, MD simulations were performed on mutated cetuximab at the residues listed above. RMSD values are reported in Supplementary Figure S4.

**Figure 8:**
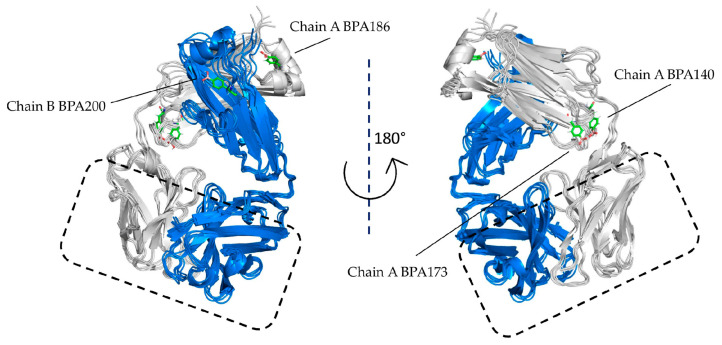
Comparison of protein structures: 3D superimposition of representative structures obtained from cluster analysis shows structural similarity among wild type and boronated proteins, especially in the EGFR interaction region.

The comparison between wild type and boronated residues was performed using RMSD analysis. Both wild type (Tyr140, 173, 186, and 200) and corresponding boronated residues stabilized at an average of about 1.5 Å in both chains (Figure S4). The similar RMDS trends for wild type and boronated protein suggested a negligible effect on structural changes determined by the boronated residue insertion. A structural analysis between wild type and boronated residues was successively performed using cluster analysis to highlight minimal differences in protein rearrangement. RMSF values were calculated and the corresponding plots are reported in Supplementary Figure S5. Comparing the wild type protein chains with those that were boronated, RMSF values remained almost equally fluctuating. In addition, clustering analysis was performed on each trajectory to capture the representative conformation of wild type and boronated residues of the monoclonal antibody. Clusters with the highest population were distributed in the following way: 30% for wild type, 77% for Tyr140, 38% for Tyr173, 72% for Tyr186 and 70% for Tyr200. Representative structures for each cluster were chosen to compare the conformational changes among wild type and corresponding boronated residues. As shown in Figure 8, the native overall structure was maintained in the boronated protein, and as was foreseeable, the monoclonal antibody interaction region with EGFR was fully preserved.

Moreover, the total number of hydrogen bonds of wild type and boronated residues were also conserved. In fact, the analysis of the hydrogen bonds along the trajectories for both wild type and boronated proteins indicates a total hydrogen bond number of about 350, while the boronated residues made the same hydrogen bonds as Tyr140, 173, 186, and 200, indicating a stable interaction network as in the wild type (Supplementary Figure S6 and Tables S22–S25).

## 4. Discussion and Conclusions

The results obtained from this study suggest the possibility of using boronated monoclonal antibodies in BNCT as innovative tools in anticancer therapy. The boronated antibody in fact is able to display the same selectivity in comparison with the standard monoclonal antibody, but is more powerful against cancer cells thanks to the boronation.

To develop this kind of double acting monoclonal antibody, an innovative and dedicated pipeline was implemented, capable of screening, supporting, and identifying the best amino acids that could be substituted by a boronated analogue. By doing so, particular attention was paid to potential 3D protein structure modifications and to potential steric hindrance interactions determined by the boronation. For this purpose, based on previous literature studies [13], only a suitable and small boronated moiety, namely B(OH)_2_, was inserted in specific amino acid residues. In addition, the residues far from this region were selected to preserve the mAb–target receptor interaction area and avoid the negative impact caused by steric hindrance interactions. The pipeline we developed can be used for optimizing any protein of interest as a specific interactor for cancer cells in BNCT and is based on a library of boronated compounds which display a scaffold similarity with the natural amino acid residues. The pipeline has a modular design, with an automatic flux of data in the boronation simulation, e.g., evaluation of the most suitable residue types and residue positions to be boronated, evaluation of the monoclonal antibody folding after insertion of the specific boronated residue, and ranking of the best results after boronation using a Python script.

The pipeline applied to the monoclonal antibody cetuximab, as a case study, allowed us to identify by means of molecular docking four Tyr residues as the best to be mutated, among the several present. The similarity of Tyr with phenyl boronic acid, *p*-toluene boronic acid, and cyclopentil boronic acid relies on similar steric and polar features. In fact, the only hydrophobic feature (e.g., an aliphatic (Hys) or an aromatic ring (Phe, Tyr, and Trp)) is insufficient by itself to perform the best pocket occupancy, and the polar component (e.g., OH, nitrogen atom) should integrate it. MD simulations proved to be a very efficient tool to evaluate the correct protein folding, letting us predict whether the mutations impact the 3D mAb structure. Specifically, the four Tyr residues suggested by these docking studies and confirmed by MD simulations were, among the others, the best capable of retaining the native protein folding and guaranteed the high binding specificity of cetuximab to EGFR.

In the past, boronated mAbs were prepared using large boron-containing molecules or dendrimers, but the boronated antibody specificity and structural and functional features were not preserved.

In this context, we developed a pipeline useful for a fast and precise evaluation of the effect of boronated modification on the 3D structure of monoclonal antibodies. The developed pipeline was tested on cetuximab, inserting an increased number of boron items without consequent conformational changes of the mAb. The preservation of the mAb 3D structure ensures the mAb specificity and strength in the binding to target. The protocol can be generalized and applied to any monoclonal antibody used in cancer therapy. In the present work, cetuximab, a chimeric monoclonal antibody capable of inhibiting EGFR and decelerating tumor growth, has been discussed as a case study. Of note, the expression of EGFR is estimated to be around 0.5–1 × 10^5^ per each normal cell [28], and it is overexpressed 10^6^ times more per cancer cell [29]. Thus, it is potentially possible to achieve more than 10^9 10^B atom per cancer cell.

Thus, the boronated mAb can perform double anti-tumor activity: chemotherapy, related to its typical action, and radiotherapy, as a sort of boost, due to the neutron irradiation on ^10^B. The mAb could be obtained based on unnatural amino acid technology, using tyrosine building block 4-borono-L-phenylalanine and applying a solid phase synthesis using an automated peptide synthesizer [30].

In conclusion, based on these findings, this innovative computational pipeline and this application on cetuximab as a case study provide evidence that BNCT treatment can benefit from the experience of using Monoclonal Antibodies as anti-tumor drugs; specifically, mAbs are suitable tools to drive boron on tumor targets.

## Data Availability

Not applicable.

## Supplementary Materials

The following are available online at https://www.mdpi.com/article/10.3390/cells10113225/s1.
