# Supplementary material for: A Boron Delivery Antibody (BDA) with Boronated Specific Residues: New Perspectives in Boron Neutron Capture Therapy from an In Silico Investigation
Source: Cells. 2021 Nov 18;10(11):3225. doi: 10.3390/cells10113225 (PMC8618907; doi:10.3390/cells10113225)
Supplement: Supplementary file 1 [file cells-10-03225-s001.zip › cells-1458144-supplementary.pdf]

SUPPLEMENTARY MATERIAL

A Boron Delivery Antibody (BDA) with boronated specific residues: new perspectives in Boron Neutron Capture Therapy.

Rondina A.[1$], Fossa P.[2$], Orro A.[1], Milanesi L.[1], De Palma A.[1], Perico D.[1], Mauri P.[1*] and D'Ursi P.[1*]

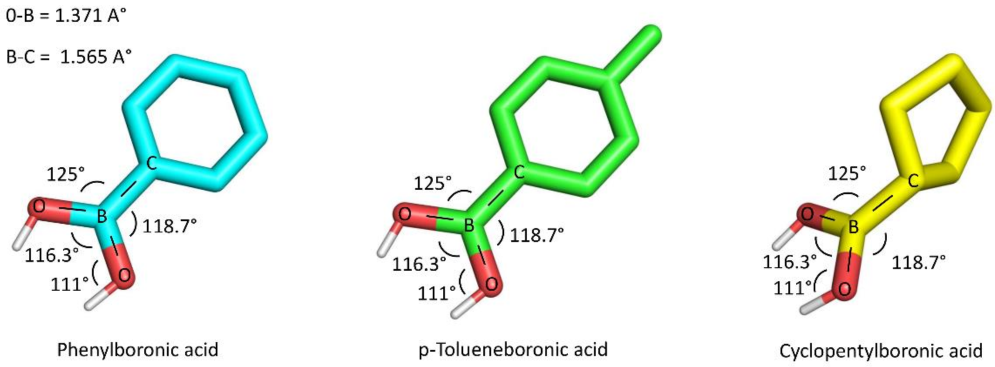

Supplementary Figure S1. Fragment probes parametrization. Carbon-boron-oxygen and boron-oxygen angle degrees and carbon -boron and boron-oxygen bond lengths are reported for each fragment probe.

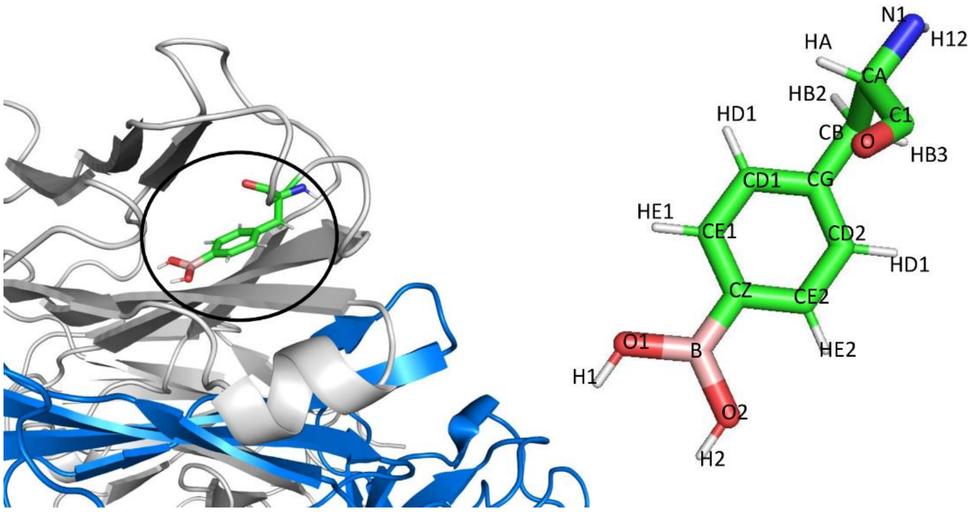

Supplementary Figure S2. Chain A BPA186. Graphical representation of BPA inserted within Cetuximab (left). Atom names of the new modified residue (right).

Supplementary Table S1. Bond length: atom name referred to Supplementary Figure 2.

Bond	Å
O1-B	1.371
O2-B	1.371
B-CZ	1.565

Supplementary Table S2. Angles: atom names referred to Supplementary Figure 2.

Angle	(◦)
O1-B-CZ	125
CZ-B-O2	118.7
O1-B-O2	116.3
B-O1-H1	111
B-O2-H2	111

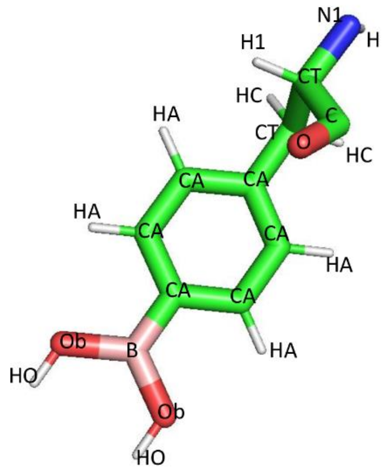

Supplementary Figure S3. BPA atom names used for parameterization in Amber18 Molecular Dynamics package.

Supplementary Table S3. Bond parameters (*This study, ** Ref [18]).

Bond	Kr (kcal (mol·Å^2) $^{-1}$)	req (Å)	Source
H -N	434.00	1.010	*
CT-N	337.00	1.449	*
CT-CT	310.00	1.526	*
CT-H1	340.00	1.090	*
C -CT	317.00	1.522	*
CA-CT	317.00	1.510	*
CT-HC	340.00	1.090	*
CA-CA	469.00	1.400	*
CA-HA	367.00	1.080	*
C -O	570.00	1.229	*
B -CA	218.951	1.559	**
HO-Ob	546.139	0.970	**
Ob-B	350.221	1.372	**

Supplementary Table S4. Angle parameters (*This study, ** Ref [18]).

Angle	Kθ (kcal/(mol·radian²))	θeq (◦)	Source
CT-CT-N	80.000	109.700	*
H1-CT-N	50.000	109.500	*
C -CT-N	63.000	110.100	*
CT-N -H	50.000	118.040	*
C -CT-H1	50.000	109.500	*
CA-CT-CT	63.000	114.000	*
CT-CT-HC	50.000	109.500	*
CT-C -O	80.000	120.400	*
CT-CT-H1	50.000	109.500	*
C -CT-CT	63.000	111.100	*
HC-CT-HC	35.000	109.500	*
CA-CA-CT	70.000	120.000	*
CA-CT-HC	50.000	109.500	*
CA-CA-CA	63.000	120.000	*
CA-CA-HA	50.000	120.000	*
CA-CA-B	127.380	120.970	**
CA-B -Ob	89.005	118.100	**
HO-Ob-B	50.184	110.550	**
Ob-B -Ob	99.046	122.400	**

Supplementary Table S5. Dihedral parameters (*This study, ** Ref [18]).

Dihedral	Divider	Vn (kcal/ mol)	γ	n	Source
CA-CT-CT-N	9	1.400	0.000	3.000	*
HC-CT-CT-N	9	1.400	0.000	3.000	*
O -C -CT-N	6	0.000	0.000	2.000	*
CT-CT-N -H	6	0.000	0.000	2.000	*
H1-CT-N -H	6	0.000	0.000	2.000	*
C -CT-N -H	6	0.000	0.000	2.000	*
CA-CA-CT-CT	6	0.000	0.000	2.000	*
O -C -CT-CT	6	0.000	0.000	2.000	*
CA-CA-CA-CT	4	4.500	180.000	2.000	*
CT-CA-CA-HA	4	4.500	180.000	2.000	*
CA-CT-CT-H1	9	1.400	0.000	3.000	*
C -CT-CT-CA	9	1.400	0.000	3.000	*
CA-CA-CA-CA	4	4.500	180.000	2.000	*
CA-CA-CA-HA	4	4.500	180.000	2.000	*
CA-CA-CT-HC	6	0.000	0.000	2.000	*
HA-CA-CA-HA	4	4.500	180.000	2.000	*
H1-CT-CT-HC	9	1.400	0.000	3.000	*
C -CT-CT-HC	9	1.400	0.000	3.000	*
O -C -CT-H1	1	0.800	0.000	-1.000	*
O -C -CT-H1	1	0.000	0.000	-2.000	*
O -C -CT-H1	1	0.080	180.000	3.000	*
CA-CA-B -Ob	1	0.500	180.000	2.000	**

HO-Ob-B -CA	1	2.700	180.000	2.000	**
HO-Ob-B -Ob	1	2.700	180.000	2.000	**
Ob-B -Ob-HO	1	2.700	180.000	2.000	**

Supplementary Table S6. Blind docking results for Phe mutated to Gly, fragment probe *p*-toluene boronic acid.

pose number	affinity (kcal/mol)	nearest Phe (in WT protein)	Distance (Å)	Angle (°)
1	-5.60	Chain B Phe128	4.19	135.49
2	-5.20	Chain B Phe128	2.26	45.78
3	-5.10	Chain A Phe209	1.06	112.90
4	-5.00	Chain B Phe106	13.90	111.47
5	-5.00	Chain B Phe106	13.90	111.09
6	-4.90	Chain B Phe128	10.15	99.44
7	-4.80	Chain A Phe98	12.61	87.51
8	-4.80	Chain B Phe172	18.47	79.41
9	-4.60	Chain B Phe106	14.78	104.37
10	-4.60	Chain B Phe128	9.19	78.61
11	-4.60	Chain A Phe21	13.47	133.27
12	-4.50	Chain B Phe172	13.57	133.33
13	-4.50	Chain B Phe152	10.93	104.66
14	-4.50	Chain B Phe152	4.57	127.16
15	-4.50	Chain A Phe98	13.36	119.93
16	-4.40	Chain B Phe80	14.54	133.36
17	-4.40	Chain A Phe139	14.94	67.23
18	-4.30	Chain B Phe63	12.98	111.00
19	-4.30	Chain B Phe152	13.91	112.21
20	-4.20	Chain B Phe172	10.11	99.86

Supplementary Table S7. Blind docking results for Phe mutated to Gly, fragment probe cyclopentilboronic acid.

pose number	affinity (kcal/mol)	nearest Phe (in WT protein)	Distance (Å)	Angle (°)
1	-5.40	Chain A Phe98	3.95	135.65
2	-4.90	Chain B Phe106	2.79	107.63
3	-4.50	Chain B Phe106	2.19	63.48
4	-4.40	Chain A Phe98	12.29	32.07
5	-4.10	Chain B Phe172	17.74	69.00
6	-4.10	Chain B Phe152	2.93	123.62
7	-4.00	Chain B Phe106	12.06	92.30
8	-3.90	Chain B Phe152	10.79	94.58
9	-3.90	Chain B Phe172	13.98	126.45
10	-3.80	Chain B Phe152	4.58	154.87
11	-3.80	Chain B Phe172	14.48	98.93
12	-3.80	Chain B Phe106	13.58	51.76
13	-3.80	Chain B Phe152	14.39	31.79

14	-3.80	Chain A Phe21	12.04	101.71
15	-3.70	Chain B Phe172	14.04	80.85
16	-3.60	Chain B Phe172	10.07	74.32
17	-3.60	Chain B Phe79	3.50	144.92
18	-3.60	Chain A Phe116	14.10	93.56
19	-3.50	Chain B Phe152	14.46	95.41
20	-3.40	Chain B Phe172	10.08	97.49

Supplementary Table S8. Blind docking results for Phe mutated to Ala, fragment probe phenylboronic acid.

pose number	affinity (kcal/mol)	nearest Phe (in WT protein)	Distance (Å)	Angle (°)
1	-4.80	Chain B Phe128	3.03	65.95
2	-4.80	Chain A Phe98	12.32	38.52
3	-4.70	Chain B Phe128	10.13	99.39
4	-4.60	Chain B Phe106	13.94	112.47
5	-4.50	Chain B Phe172	18.52	78.22
6	-4.40	Chain B Phe152	14.42	69.73
7	-4.40	Chain B Phe106	13.66	25.79
8	-4.40	Chain A Phe209	7.36	86.94
9	-4.40	Chain A Phe21	13.75	133.24
10	-4.30	Chain A Phe98	12.11	72.53
11	-4.30	Chain A Phe209	5.30	26.12
12	-4.20	Chain B Phe152	13.53	80.00
13	-4.20	Chain A Phe98	8.80	30.37
14	-4.20	Chain B Phe27	14.28	65.03
15	-4.00	Chain B Phe172	10.15	128.92
16	-4.00	Chain B Phe106	10.09	144.34
17	-3.80	Chain B Phe172	7.83	139.84
18	-3.80	Chain B Phe128	17.20	118.44
19	-3.80	Chain B Phe106	12.65	55.98
20	-3.80	Chain B Phe152	13.91	92.01

Supplementary Table S9. Blind docking results for Phe mutated to Ala, fragment probe cyclopentilboronic acid.

pose number	affinity (kcal/mol)	nearest Phe (in WT protein)	Distance (Å)	Angle (°)
1	-4.30	Chain B Phe106	14.67	118.69
2	-4.20	Chain B Phe128	9.64	88.90
3	-4.20	Chain A Phe139	9.71	124.89
4	-4.10	Chain A Phe98	12.57	26.08
5	-4.10	Chain A Phe98	14.55	43.43
6	-4.10	Chain A Phe21	13.43	119.53
7	-4.00	Chain B Phe106	12.01	92.78
8	-4.00	Chain B Phe172	17.54	84.77
9	-4.00	Chain B Phe172	14.99	85.75
10	-4.00	Chain A Phe98	7.97	30.86
11	-3.90	Chain A Phe98	13.45	117.13

12	-3.90	Chain A Phe98	12.47	57.51
13	-3.90	Chain B Phe152	10.71	94.16
14	-3.80	Chain B Phe128	9.70	86.54
15	-3.80	Chain B Phe80	10.36	108.60
16	-3.80	Chain A Phe98	12.73	110.16
17	-3.80	Chain A Phe139	16.92	100.41
18	-3.80	Chain A Phe98	14.01	130.68
19	-3.80	Chain A Phe139	18.49	102.19
20	-3.80	Chain B Phe152	13.41	91.58

Supplementary Table S10. Blind docking results for Tyr mutated to Gly, fragment probe *p*-toluene boronic acid.

pose number	affinity (kcal/mol)	nearest Phe (in WT protein)	Distance (Å)	Angle (°)
1	-5.80	Chain A Tyr173	3.87	129.31
2	-5.40	Chain B Tyr94	1.68	38.31
3	-5.40	Chain A Tyr173	2.23	61.76
4	-5.30	Chain A Tyr173	1.80	55.81
5	-5.30	Chain A Tyr140	5.14	154.86
6	-5.30	Chain A Tyr173	3.50	83.84
7	-5.20	Chain A Tyr173	3.62	14.74
8	-5.00	Chain A Tyr186	13.85	109.12
9	-5.00	Chain B Tyr59	15.18	147.16
10	-4.90	Chain B Tyr104	4.91	159.48
11	-4.80	Chain B Tyr200	5.52	118.14
12	-4.80	Chain B Tyr151	14.24	130.90
13	-4.70	Chain B Tyr182	3.96	106.03
14	-4.50	Chain B Tyr200	5.61	141.93
15	-4.40	Chain A Tyr87	11.39	155.55
16	-4.40	Chain B Tyr200	12.45	105.15
17	-4.40	Chain B Tyr182	1.65	65.12
18	-4.40	Chain B Tyr182	10.59	74.00
19	-4.40	Chain B Tyr182	4.21	109.78
20	-4.30	Chain B Tyr182	10.34	86.85

Supplementary Table S11. Blind docking results for Tyr mutated to Gly, fragment probe cyclopentilboronic acid.

pose number	affinity (kcal/mol)	nearest Phe (in WT protein)	Distance (Å)	Angle (°)
1	-5.10	Chain B Tyr94	1.67	50.75
2	-4.80	Chain B Tyr94	3.24	75.19
3	-4.60	Chain A Tyr186	2.24	80.13
4	-4.60	Chain A Tyr173	5.43	135.78
5	-4.60	Chain A Tyr192	2.26	86.59
6	-4.50	Chain B Tyr94	2.24	31.05
7	-4.50	Chain A Tyr140	4.79	157.82
8	-4.40	Chain A Tyr173	1.88	11.24
9	-4.30	Chain A Tyr186	2.70	114.18

10	-4.30	Chain A Tyr192	2.37	67.09
11	-4.20	Chain A Tyr140	3.44	98.78
12	-4.20	Chain B Tyr151	13.80	117.54
13	-4.20	Chain A Tyr140	2.91	121.08
14	-4.20	Chain A Tyr140	5.07	99.50
15	-4.10	Chain A Tyr140	5.44	135.87
16	-4.00	Chain A Tyr87	9.68	120.89
17	-3.90	Chain B Tyr93	14.32	46.04
18	-3.90	Chain B Tyr182	1.57	49.30
19	-3.80	Chain A Tyr140	1.50	65.13
20	-3.70	Chain B Tyr94	10.28	152.16

Supplementary Table S12. Blind docking results for Tyr mutated to Ala, fragment probe phenylboronic acid.

pose number	affinity (kcal/mol)	nearest Phe (in WT protein)	Distance (Å)	Angle (°)
1	-5.30	Chain A Tyr192	2.22	81.05
2	-5.10	Chain A Tyr140	7.62	136.46
3	-5.10	Chain A Tyr192	2.14	81.08
4	-4.80	Chain A Tyr173	2.48	66.41
5	-4.80	Chain A Tyr173	3.47	70.56
6	-4.70	Chain A Tyr186	13.86	109.09
7	-4.70	Chain A Tyr173	2.49	10.07
8	-4.70	Chain B Tyr93	15.02	55.02
9	-4.60	Chain A Tyr140	3.06	40.90
10	-4.60	Chain A Tyr186	1.91	18.49
11	-4.60	Chain B Tyr200	5.50	116.97
12	-4.60	Chain A Tyr140	4.29	141.66
13	-4.50	Chain A Tyr87	12.67	128.60
14	-4.30	Chain A Tyr87	13.61	117.97
15	-4.30	Chain A Tyr87	11.49	166.07
16	-4.20	Chain B Tyr200	6.90	150.40
17	-4.20	Chain A Tyr86	12.46	136.99
18	-4.20	Chain A Tyr87	10.06	83.51
19	-4.20	Chain B Tyr182	10.60	73.29
20	-4.10	Chain A Tyr140	3.11	67.16

Supplementary Table S13. Blind docking results for Tyr mutated to Ala, fragment probe cyclopentilboronic acid.

pose number	affinity (kcal/mol)	nearest Phe (in WT protein)	Distance (Å)	Angle (°)
1	-5.00	Chain A Tyr186	3.01	120.10
2	-4.80	Chain A Tyr173	4.03	76.30
3	-4.70	Chain A Tyr192	2.44	97.85
4	-4.40	Chain A Tyr173	2.32	11.72
5	-4.30	Chain A Tyr140	3.66	117.56
6	-4.30	Chain A Tyr140	4.84	123.18
7	-4.20	Chain B Tyr151	13.84	117.52

8	-4.10	Chain A Tyr140	5.98	132.64
9	-4.10	Chain A Tyr87	13.50	113.99
10	-4.10	Chain A Tyr173	4.00	25.64
11	-3.90	Chain B Tyr94	9.13	58.56
12	-3.90	Chain A Tyr186	2.26	51.01
13	-3.90	Chain A Tyr87	9.78	130.57
14	-3.90	Chain A Tyr87	10.85	166.51
15	-3.90	Chain B Tyr93	9.35	148.60
16	-3.80	Chain A Tyr192	15.92	83.81
17	-3.80	Chain A Tyr87	10.78	120.11
18	-3.80	Chain A Tyr 87	8.72	118.57
19	-3.80	Chain B Tyr200	1.93	68.88
20	-3.70	Chain B Tyr94	10.25	152.44

Supplementary Table S14. Blind docking results for Trp mutated to Gly, fragment probe *p*-toluene boronic acid.

pose number	affinity (kcal/mol)	nearest Phe (in WT protein)	Distance (Å)	Angle (°)
1	-6.50	Chain B Trp109	4.87	122.28
2	-5.60	Chain B Trp36	3.32	89.74
3	-5.50	Chain A Trp94	3.97	64.97
4	-5.20	Chain A Trp148	3.47	112.14
5	-5.20	Chain B Trp47	2.23	76.88
6	-5.00	Chain B Trp160	17.64	61.51
7	-4.90	Chain B Trp36	12.71	147.37
8	-4.80	Chain B Trp52	1.50	73.32
9	-4.80	Chain A Trp148	16.46	118.88
10	-4.80	Chain B Trp160	11.61	78.24
11	-4.60	Chain B Trp109	14.68	62.13
12	-4.60	Chain A Trp35	18.02	75.78
13	-4.60	Chain B Trp109	17.78	131.32
14	-4.60	Chain B Trp109	12.49	31.46
15	-4.50	Chain B Trp109	14.97	87.63
16	-4.50	Chain A Trp35	16.29	16.42
17	-4.40	Chain A Trp35	16.18	116.23
18	-4.40	Chain A Trp94	8.42	130.99
19	-4.40	Chain B Trp47	8.43	115.86
20	-4.30	Chain B Trp109	11.92	29.55

Supplementary Table S15. Blind docking results for Trp mutated to Gly, fragment probe cyclopentilboronic acid.

pose number	affinity (kcal/mol)	nearest Phe (in WT protein)	Distance (Å)	Angle (°)
1	-5.10	Chain B Trp47	4.60	101.61
2	-4.20	Chain A Trp148	15.26	97.15
3	-4.20	Chain B Trp52	4.09	142.24
4	-4.20	Chain B Trp47	2.99	82.92
5	-4.20	Chain B Trp52	1.58	61.79

6	-4.10	Chain B Trp160	10.77	89.48
7	-4.10	Chain B Trp109	13.52	110.31
8	-4.10	Chain B Trp109	12.40	106.13
9	-4.00	Chain B Trp109	17.91	119.05
10	-4.00	Chain B Trp47	7.34	127.11
11	-3.80	Chain B Trp36	14.51	96.09
12	-3.80	Chain B Trp109	15.14	22.60
13	-3.80	Chain B Trp109	15.33	46.91
14	-3.80	Chain B Trp109	14.55	109.40
15	-3.80	Chain B Trp47	9.13	23.32
16	-3.70	Chain B Trp36	13.63	58.01
17	-3.70	Chain B Trp160	18.17	82.94
18	-3.70	Chain B Trp109	13.32	48.23
19	-3.60	Chain B Trp160	12.26	66.57
20	-3.60	Chain B Trp109	14.06	73.74

Supplementary Table S16. Blind docking results for Trp mutated to Ala, fragment probe phenylboronic acid.

pose number	affinity (kcal/mol)	nearest Phe (in WT protein)	Distance (Å)	Angle (°)
1	-6.70	Chain B Trp109	5.21	103.46
2	-5.70	Chain A Trp94	4.90	75.20
3	-5.20	Chain B Trp109	5.31	151.94
4	-5.00	Chain A Trp94	3.03	45.12
5	-4.90	Chain A Trp148	15.69	89.71
6	-4.70	Chain B Trp160	17.60	60.90
7	-4.70	Chain B Trp36	12.73	146.91
8	-4.70	Chain B Trp109	13.10	102.78
9	-4.50	Chain B Trp160	11.62	77.81
10	-4.40	Chain B Trp109	13.16	53.43
11	-4.40	Chain A Trp35	18.25	66.91
12	-4.30	Chain B Trp109	14.08	111.01
13	-4.30	Chain B Trp160	12.30	94.20
14	-4.20	Chain A Trp35	16.36	17.75
15	-4.20	Chain A Trp35	15.59	129.87
16	-4.10	Chain B Trp52	4.31	172.29
17	-4.10	Chain B Trp36	14.10	89.20
18	-4.10	Chain B Trp36	14.21	61.44
19	-4.00	Chain B Trp160	15.79	73.41
20	-4.00	Chain B Trp160	17.38	171.00

Su pplementary Table S17. Blind docking results for Trp mutated to Ala, fragment probe cyclopentilboronic acid.

pose number	affinity (kcal/mol)	nearest Phe (in WT protein)	Distance (Å)	Angle (°)
1	-4.40	Chain B Trp52	2.51	75.69
2	-4.30	Chain B Trp160	17.61	57.22
3	-4.20	Chain B Trp109	12.64	110.66

4	-4.20	Chain B Trp160	10.80	87.76
5	-4.10	Chain B Trp109	13.30	107.29
6	-4.00	Chain B Trp109	17.93	118.77
7	-3.90	Chain A Trp35	15.68	62.59
8	-3.90	Chain B Trp47	9.24	35.32
9	-3.90	Chain A Trp94	10.08	114.68
10	-3.80	Chain B Trp160	13.04	10.23
11	-3.80	Chain B Trp109	14.50	110.20
12	-3.80	Chain B Trp47	10.59	89.18
13	-3.70	Chain B Trp109	15.12	25.80
14	-3.70	Chain B Trp109	14.72	91.03
15	-3.70	Chain B Trp109	12.21	9.59
16	-3.60	Chain B Trp160	15.68	59.08
17	-3.60	Chain B Trp160	13.68	49.29
18	-3.60	Chain B Trp36	12.50	150.50
19	-3.50	Chain A Trp148	8.07	95.21
20	-3.40	Chain A Trp148	11.28	63.95

Supplementary Table S18. Blind docking results for His mutated to Gly, fragment probe *p*-toluene boronic acid.

pose number	affinity (kcal/mol)	nearest Phe (in WT protein)	Distance (Å)	Angle (°)
1	-5.00	Chain A His189	20.86	56.59
2	-5.00	Chain B His35	18.42	66.14
3	-5.00	Chain B His35	9.97	87.65
4	-4.80	Chain B His206	18.28	136.59
5	-4.80	Chain B His170	10.95	110.06
6	-4.70	Chain A His189	5.26	62.99
7	-4.60	Chain B His206	14.88	91.62
8	-4.60	Chain B His206	19.04	81.73
9	-4.60	Chain B His206	13.28	75.18
10	-4.50	Chain A His189	11.03	72.77
11	-4.50	Chain A His189	14.31	89.69
12	-4.50	Chain A His189	15.84	111.04
13	-4.50	Chain B His206	18.97	53.65
14	-4.40	Chain B His35	12.69	98.15
15	-4.40	Chain A His189	15.62	112.59
16	-4.40	Chain B His35	20.36	127.20
17	-4.40	Chain B His170	5.69	83.96
18	-4.30	Chain B His170	16.60	76.95
19	-4.00	Chain B His170	13.49	60.40
20	-4.00	Chain A His34	15.04	141.80

Supplementary Table S19. Blind docking results for His mutated to Gly, fragment probe cyclopentilboronic acid.

pose number	affinity (kcal/mol)	nearest Phe (in WT protein)	Distance (Å)	Angle (°)
1	-4.20	Chain B His206	17.99	161.73

2	-4.20	Chain B His35	10.68	121.61
3	-4.20	Chain B His206	10.82	123.70
4	-4.20	Chain A His34	14.49	50.00
5	-4.10	Chain B His170	3.96	72.23
6	-4.10	Chain B His206	18.00	166.24
7	-4.10	Chain B His206	17.67	20.97
8	-4.00	Chain B His170	18.89	58.10
9	-4.00	Chain B His35	18.31	59.82
10	-3.90	Chain B His206	11.24	88.04
11	-3.80	Chain A His34	18.90	44.99
12	-3.80	Chain B His206	15.65	77.65
13	-3.80	Chain B His35	9.90	116.10
14	-3.80	Chain B His35	10.61	117.27
15	-3.70	Chain A His189	13.05	59.97
16	-3.70	Chain B His170	11.90	146.89
17	-3.60	Chain B His170	15.34	85.93
18	-3.60	Chain A His189	12.95	149.20
19	-3.60	Chain B His170	15.94	77.64
20	-3.50	Chain A His189	19.68	116.09

Supplementary Table S20. Blind docking results for His mutated to Ala, fragment probe phenylboronic acid.

pose number	affinity (kcal/mol)	nearest Phe (in WT protein)	Distance (Å)	Angle (°)
1	-4.70	Chain A His189	20.87	56.46
2	-4.70	Chain B His35	19.33	77.66
3	-4.70	Chain B His35	18.47	65.48
4	-4.60	Chain B His206	18.83	155.78
5	-4.60	Chain B His170	5.28	58.52
6	-4.60	Chain A His189	16.36	103.62
7	-4.50	Chain B His206	10.99	109.43
8	-4.40	Chain B His 206	11.93	100.20
9	-4.30	Chain B His 206	14.86	93.30
10	-4.30	Chain A His189	12.90	157.11
11	-4.10	Chain A His189	18.22	39.57
12	-4.10	Chain A His189	17.29	104.73
13	-4.00	Chain B His206	14.65	160.47
14	-4.00	Chain B His35	16.02	55.41
15	-4.00	Chain A His189	2.21	88.68
16	-4.00	Chain B His35	18.36	128.47
17	-4.00	Chain B His170	9.36	53.77
18	-3.90	Chain B His170	6.41	49.46
19	-3.90	Chain B His170	22.32	43.84
20	-3.80	Chain A His34	12.85	101.20

Supplementary Table S21. Blind docking results for His mutated to Ala, fragment probe cyclopentilboronic acid.

pose number	affinity (kcal/mol)	nearest Phe (in WT protein)	Distance (Å)	Angle (°)
1	-4.70	Chain B His170	4.22	47.45
2	-4.20	Chain B His206	17.93	159.58
3	-4.20	Chain B His35	10.64	121.49
4	-4.10	Chain B His206	18.25	129.73
5	-4.00	Chain A His34	13.66	25.62
6	-4.00	Chain B His35	18.03	64.52
7	-3.90	Chain B His206	13.37	45.79
8	-3.80	Chain B His170	5.29	41.99
9	-3.80	Chain B His 206	9.44	61.78
10	-3.80	Chain B His170	11.70	91.89
11	-3.80	Chain B His206	11.42	99.27
12	-3.70	Chain B His170	17.26	129.17
13	-3.70	Chain B His206	21.24	112.58
14	-3.50	Chain B His35	10.08	77.16
15	-3.50	Chain B His170	12.08	76.51
16	-3.40	Chain A His198	14.23	97.00
17	-3.40	Chain A His34	15.49	38.77
18	-3.40	Chain A His189	19.17	135.25
19	-3.40	Chain A His189	3.53	103.52
20	-3.40	Chain B His170	10.02	152.83

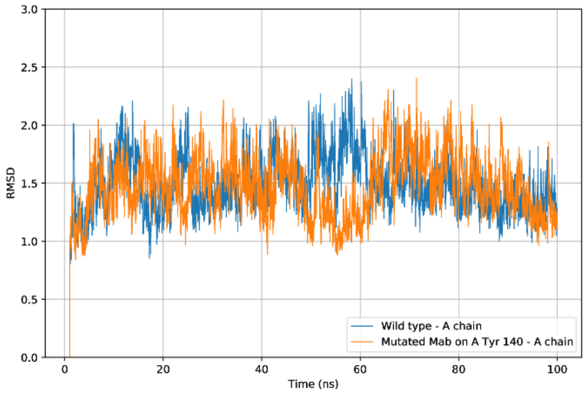

(**a**)

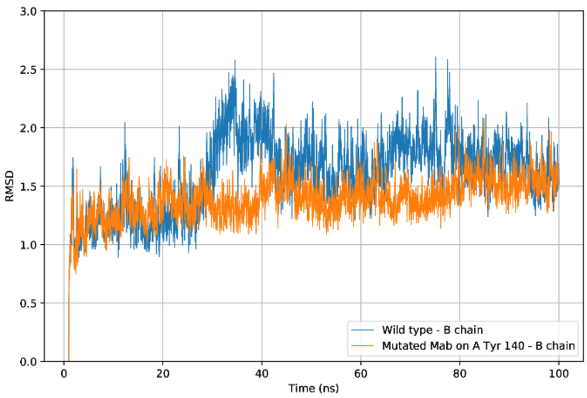

(**b**)

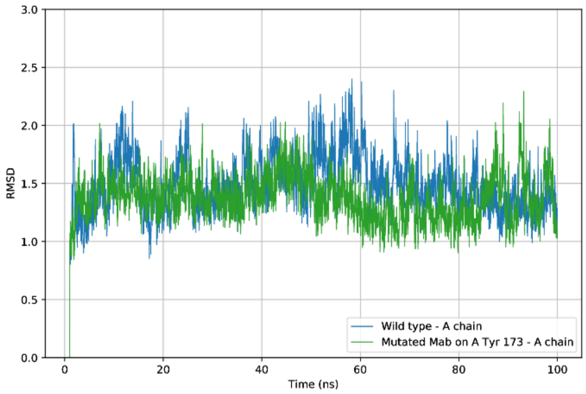

(**c**)

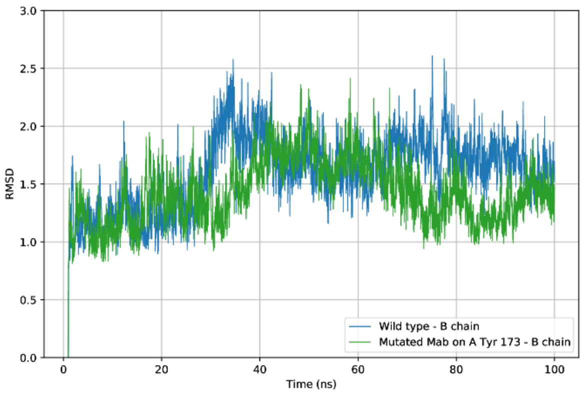

(**d**)

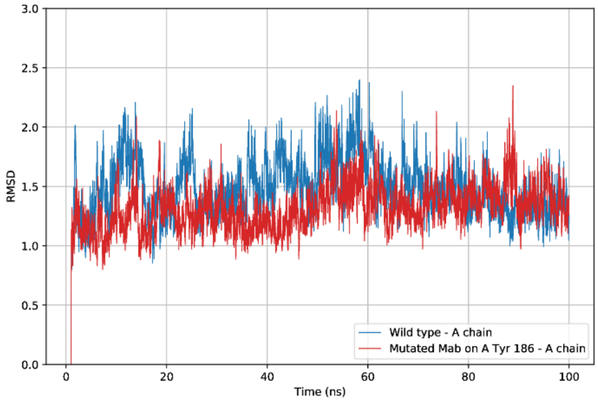

(**e**)

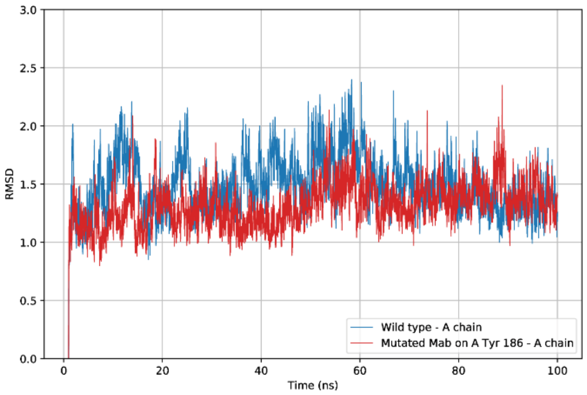

(**f**)

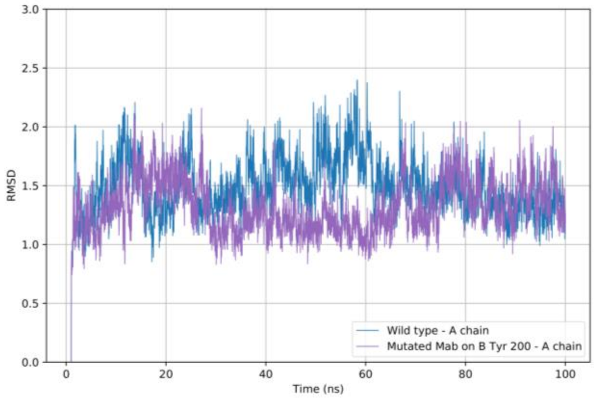

(**g**)

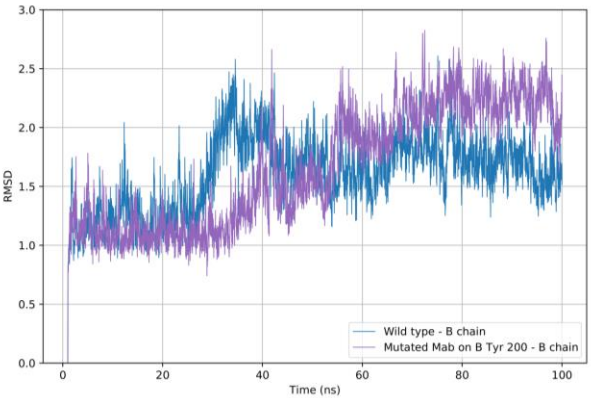

(**h**)

Supplementary Figure S4. The root means square deviation (RMSD) analysis. RMSD comparison between wild type and boronated residues on protein chain A and B. On the

left, RMSD values wild type chain A versus mutated chain A; on the right RMSD values wild type chain B and chain B after chain A (or chain B) mutation. From the top wild-type chain A (blue) and boronated Tyr140 (orange), wild type chain A (blue) and boronated Tyr173 (green), wild type chain A (blue) and boronated Tyr186 (red), wild type chain B (blue) and boronated Tyr200 (purple).

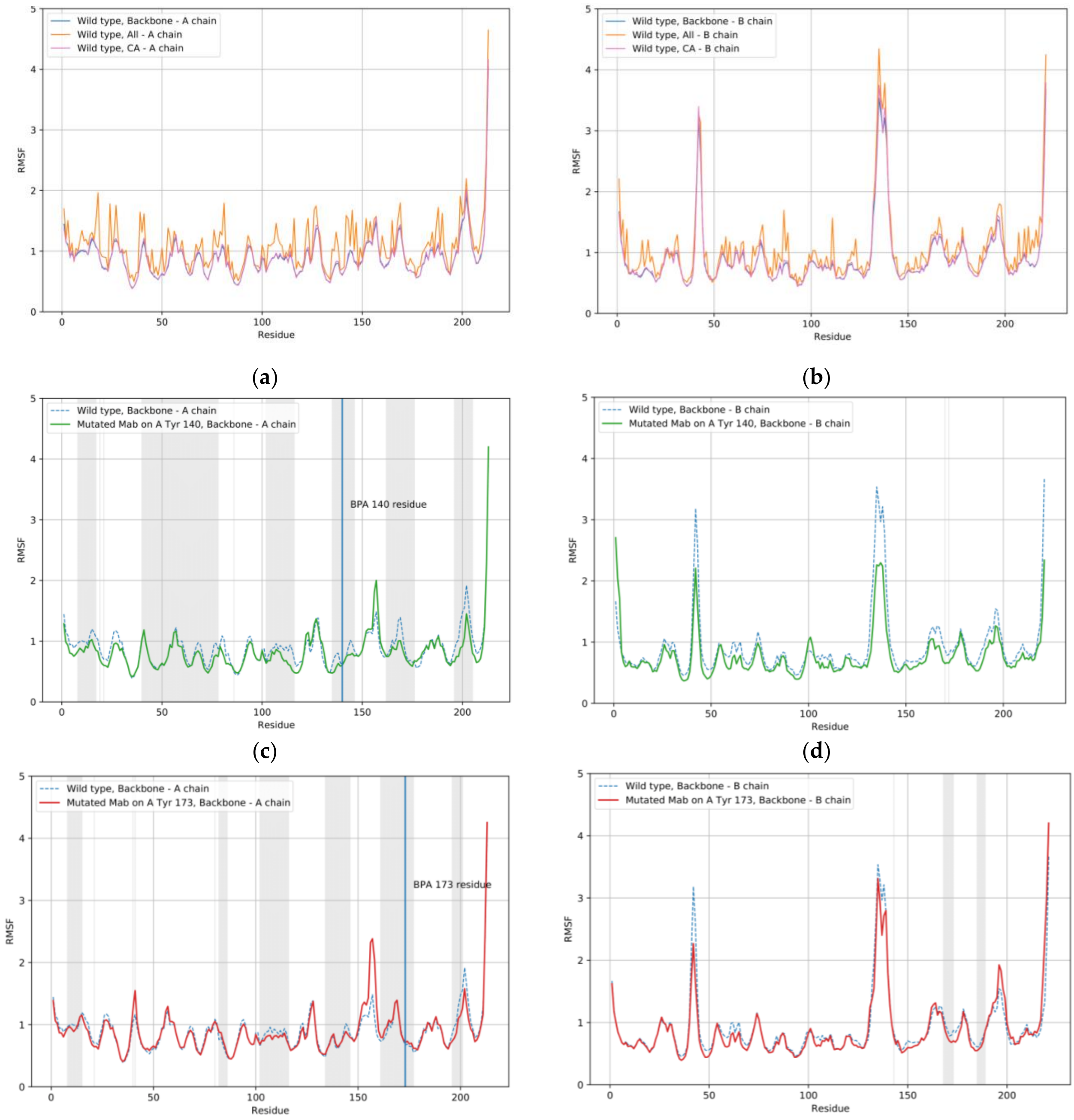

(**a**) (**b**)

(**c**) (**d**)

(**e**) (**f**)

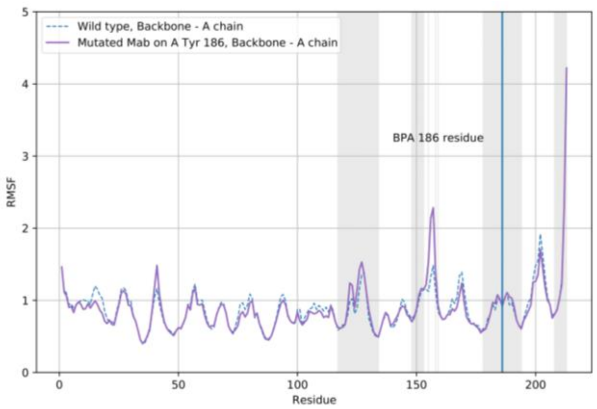

(**g**)

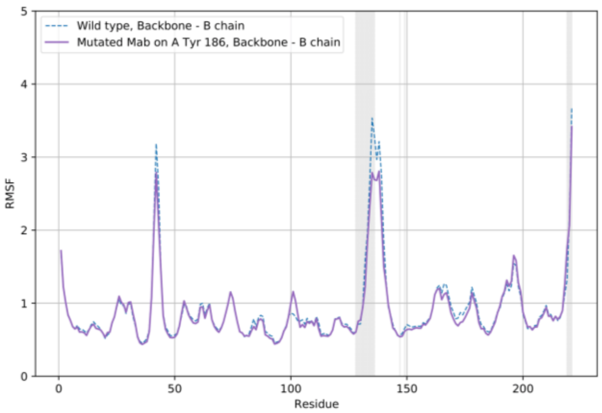

(**h**)

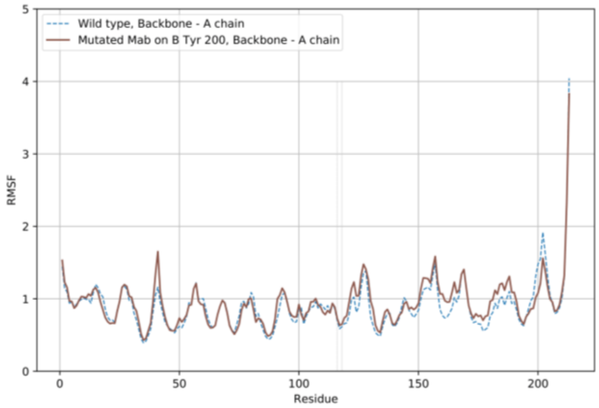

(**i**)

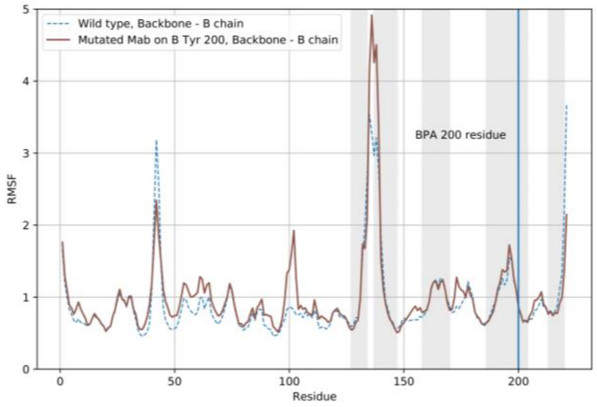

(**l**)

Supplementary Figure S5. Mean square fluctuations (RMSF) analysis, the greyed areas represent all residues within 12 Å of the mutation, while the blue vertical lines address the new mutated residue (BPA) : (**a**) RMSF calculation (total, backbone and alpha carbons) of the wild type protein (A chain); (**b**) RMSF calculation (total, backbone and alpha carbons) of the wild type protein (B chain); (**c**) Comparison of backbone fluctuations between wild type and mutated protein on residue A Tyr140 (A chain); (**d**) Comparison of backbone fluctuations between wild type and mutated protein on residue A Tyr140 (B chain); (**e**) Comparison of backbone fluctuations between wild type and mutated protein on residue A Tyr173 (A chain); (**f**) Comparison of backbone fluctuations between wild type and mutated protein on residue A Tyr173 (B chain); (**g**) Comparison of backbone fluctuations between wild type and mutated protein on residue A Tyr186 (A chain);(**h**) Comparison of backbone fluctuations between wild type and mutated protein on residue A Tyr186 (B chain); (**i**) Comparison of backbone fluctuations between wild type and mutated protein on residue B Tyr200 (A chain); (**l**) Comparison of backbone fluctuations between wild type and mutated protein on residue B Tyr200 (B chain).

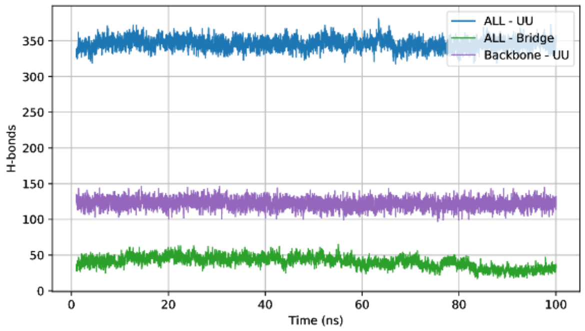

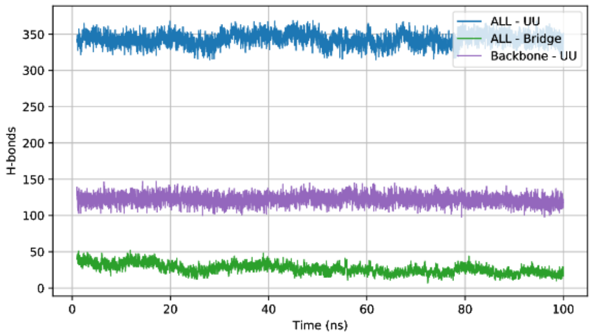

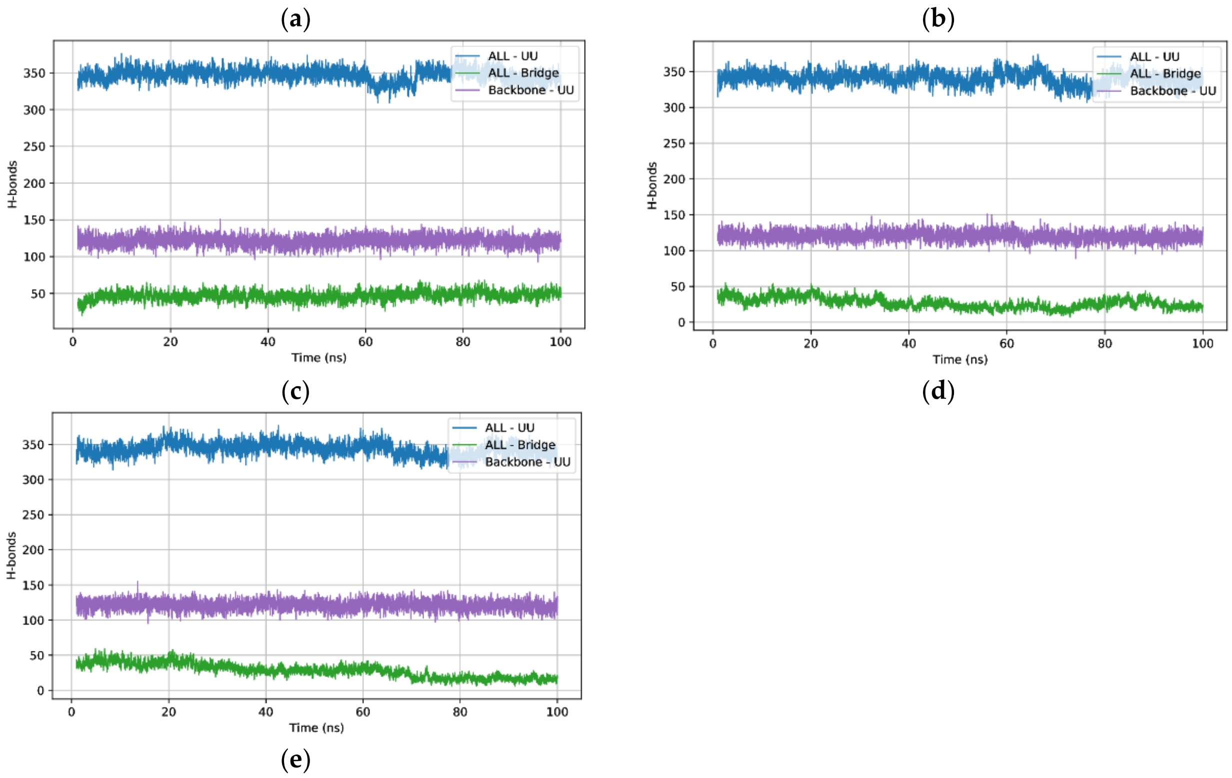

Supplementary Figure S6. H-bond analysis, comparison between the total number of hydrogen bonds in wild type Mab and mutations. the graphs show the total number of solute-solute interactions (ALL - UU), the number of Backbone UU interactions (Backbone - UU) and finally the number of hydrogen bridges (ALL - Bridge) established over the MDs simulations: (**a**) Hydrogen bonds in Wild type; (**b**) Hydrogen bonds in mutated protein (mutation on chain A Tyr140); (**c**) Hydrogen bonds in mutated protein (mutation on chain A Tyr173); (**d**) Hydrogen bonds in mutated protein (mutation on chain A Tyr186); (**e**) Hydrogen bonds in mutated protein (mutation on chain B Tyr200).

Supplementary Table S22. H-bonds analysis: comparison between the mutated residue hydrogen bonds and the counterpart residue in the wild type, lasting for more than 70% of MDs. The table below shows the donor and acceptor atom involved in the H-bond, together with information about the time fraction in which the bond lasts, the angle and the average distance of the latter.

Sample	Acceptor	Donor H	Donor	Frac	Avg Dist (Å)	Avg Ang (°)
**Wild Type**	Tyr 140/O	Ala 111/H	Ala 111/N	99%	2.96	164.27
	Ala 111/O	Tyr 140/H	Tyr 140/N	99%	2.99	161.46
**Mutated Mab on chain A Tyr140**	BPA 140/O	Ala 111/H	Ala 111/N	97%	3.00	162.67
	Ala 111/O	BPA 140/H12	BPA 140/N1	97%	3.02	165.15

Supplementary Table S23. H-bonds analysis: comparison between the mutated residue hydrogen bonds and the counterpart residue in the wild type, lasting for more than 70% of MDs. The table below shows the donor and acceptor atom involved in the H-bond, together with information about the time fraction in which the bond lasts, the angle and the average distance of the latter.

Sample	Acceptor	Donor H	Donor	Frac	Avg Dist (Å)	Avg Ang (°)
**Wild Type**	Tyr 173/O	Phe 139/H	Phe 139/N	85%	3.07	159.12
	Phe 139/O	Tyr 173/H	Tyr 173/N	95%	2.86	160.94
**Mutated Mab on chain A Tyr173**	BPA 173/O	Phe 139/H	Phe 139/N	86%	3.08	159.59
	Ala 111/O	BPA 140/H12	BPA 140/N1	97%	3.02	165.15

Supplementary Table S24. H-bonds analysis: comparison between the mutated residue hydrogen bonds and the counterpart residue in the wild type, lasting for more than 70% of MDs. The table below shows the donor and acceptor atom involved in the H-bond, together with information about the time fraction in which the bond lasts, the angle and the average distance of the latter.

Sample	Acceptor	Donor H	Donor	Frac	Avg Dist (Å)	Avg Ang (°)
**Wild Type**	Ser 182/O	Tyr 186/H	Tyr 186/N	95%	2.99	158.77
**Mutated Mab on chain A Tyr186**	Ser 182/O	BPA 186/H12	BPA 186/N1	97%	3.00	163.39

Supplementary Table S25. H-bonds analysis: comparison between the mutated residue hydrogen bonds and the counterpart residue in the wild type, lasting for more than 70% of MDs. The table below shows the donor and acceptor atom involved in the H-bond, together with information about the time fraction in which the bond lasts, the angle and the average distance of the latter.

Sample	Acceptor	Donor H	Donor	Frac	Avg Dist (Å)	Avg Ang (°)
**Wild Type**	Tyr 413/O	Val 430/H	Val 430/N	98%	2.94	161.96
	Ser 407/OG	Tyr 413/HH	Tyr 413/OH	89%	2.86	158.48
**Mutated Mab on chain B Tyr200**	BPA 413/O	Val 430/H	Val 430/N	95%	2.93	160.17
	Ser 407/OG	BPA 413/H1	BPA 413/O1	76%	2.71	162.04
